# AI Agents for Multimodal Oncology Diagnosis: Toward Transparent and Traceable Clinical Decision Support

**DOI:** 10.2196/103545

**Published:** 2026-09-15

**Authors:** Liuyang Yang, Liyu Shan, Xiangmei Yao, Renbin Zhao, Zengzheng Li, Shuai Feng, Yajie Wang

**Affiliations:** 1 Department of Hematology The First People's Hospital of Yunnan Province, Affiliated Hospital of Kunming University of Science and Technology Kunming, Yunnan China; 2 The Third Affiliated Hospital of Kunming Medical University, Yunnan Cancer Hospital, Peking University Cancer Hospital Yunnan Kunming, Yunnan China; 3 The Second Affiliated Hospital of Kunming Medical University Kunming, Yunnan China

**Keywords:** artificial intelligence agent, multimodal oncology diagnosis, clinical decision support, large language model, human–artificial intelligence collaboration, diagnostic workflow

## Abstract

Cancer diagnosis depends on data from radiology, digital pathology, molecular profiling, laboratory testing, and longitudinal clinical records. AI performs well in selected tasks, but most systems remain narrow and disconnected from the iterative reasoning required in oncology. This Viewpoint defines an AI agent as a feedback-driven system that maintains task state, selects among governed tools, observes results, and revises its plan under explicit safety constraints. This definition separates agents from multimodal foundation models, retrieval-augmented generation, and fixed workflow automation. We organize the discussion across multimodal data collection, preprocessing, fusion and representation learning, and diagnostic decision support. We distinguished agent-level evidence, component- or infrastructure-level evidence, and prospective propositions throughout. Clinical translation will require resilient failure handling, guideline version control, prospective evaluation, computational and workflow feasibility, and clinician authority over final decisions. The near-term opportunity is therefore transparent and traceable clinical decision support rather than autonomous cancer diagnosis.

## Introduction

Cancer remains a leading cause of morbidity and mortality. Global Cancer Observatory (GLOBOCAN) 2022 estimated that there were nearly 20 million new cancer cases and 9.7 million cancer-related deaths worldwide in that year [1]. Treatment delays are associated with higher mortality across several cancer indications [2]. Timely diagnostic pathways are therefore important, but they must also account for genomic, histopathological, microenvironmental, and temporal heterogeneity [3].

Modern oncology diagnosis is intrinsically multimodal. Clinicians combine imaging, pathology, laboratory results, molecular assays, and longitudinal electronic records, often across separate specialties and information systems. This fragmentation creates cognitive and coordination burdens that motivate integrated workflows [4,5]. Task-specific AI has advanced lesion detection, pathology classification, molecular modeling, and risk prediction [4,6-8]. However, most deployed or evaluated models address a single bounded input-output task rather than the full diagnostic process.

Large language models (LLMs), multimodal foundation models, and tool-use frameworks create a technical basis for coordinating these tasks [9,10]. However, a model that accepts several modalities is not automatically an agent. Similarly, retrieval-augmented generation (RAG) adds external evidence to generation, and fixed workflow orchestration links predetermined modules, but neither property alone establishes runtime planning or feedback-driven control.

This Viewpoint examines AI agents as auditable orchestration layers for multimodal oncology diagnostics. We organized the field around 4 connected stages: data collection, preprocessing, multimodal fusion and representation learning, and diagnostic decision support. Our central position is deliberately bounded: agentic orchestration may improve coordination and auditability, but clinical benefit and safety remain deployment-specific questions.

## From Task-Specific AI to Agentic Oncology Diagnostics

We used an operational definition of agentic behavior. In a conventional fixed pipeline, an output is generated through a predetermined composition, y=f_n_(...f₁(x)), and the sequence of functions does not change at runtime. An AI agent instead maintains a state *s_t_* and selects an action *a_t_* = π(g, s_t_, A_t_) from an allowed tool set *A_t_*. For a clinical goal *g*, it observes each tool’s result and updates its state before choosing the next action. The practical distinction therefore lies in conditional planning, tool selection, observation, and revision within a governed loop.

Four adjacent concepts should remain separate. A multimodal foundation model supplies reusable representations or generation across data types. RAG retrieves external sources to ground an answer. Fixed workflow orchestration executes a prescribed sequence of services. A feedback-driven AI agent can change the sequence, request missing evidence, retry or switch tools, abstain, and escalate to a clinician according to the evolving state. Long-term memory is optional and should be limited to an authorized, versioned, patient-specific or institutional state rather than treated as an unrestricted capability.

First, Figure 1 contrasts a conventional specialist synthesis with an agent-coordinated 4-stage workflow. Second, Figure 2 operationalizes the agentic layer as a plan-act-observe-update-check loop. Act coordinates the 4 stages in Figure 1B; update records state and provenance; and check evaluates evidence sufficiency, cross-modal consistency, and whether further action remains within the authorized scope. If these criteria are met, the synthesis proceeds to clinician review. Remediable gaps return the workflow to planning; gaps that cannot be supplemented trigger clinician escalation. The agent-coordinated elements in both figures are prospective design propositions and not validated clinical systems or performance claims.

We applied 3 evidence labels throughout this Viewpoint. Agent-level evidence evaluates a complete loop that plans, uses tools, and produces an integrated output, as in the external oncology evaluation by Ferber et al [11]. Component or infrastructure evidence evaluates a model, retrieval method, data pipeline, or governance mechanism that an agent could invoke [12]. Prospective propositions describe plausible clinical workflows that still require system-level and prospective validation. This hierarchy prevents strong component performance from being misread as proof of a clinically validated agent.

**Figure 1:**
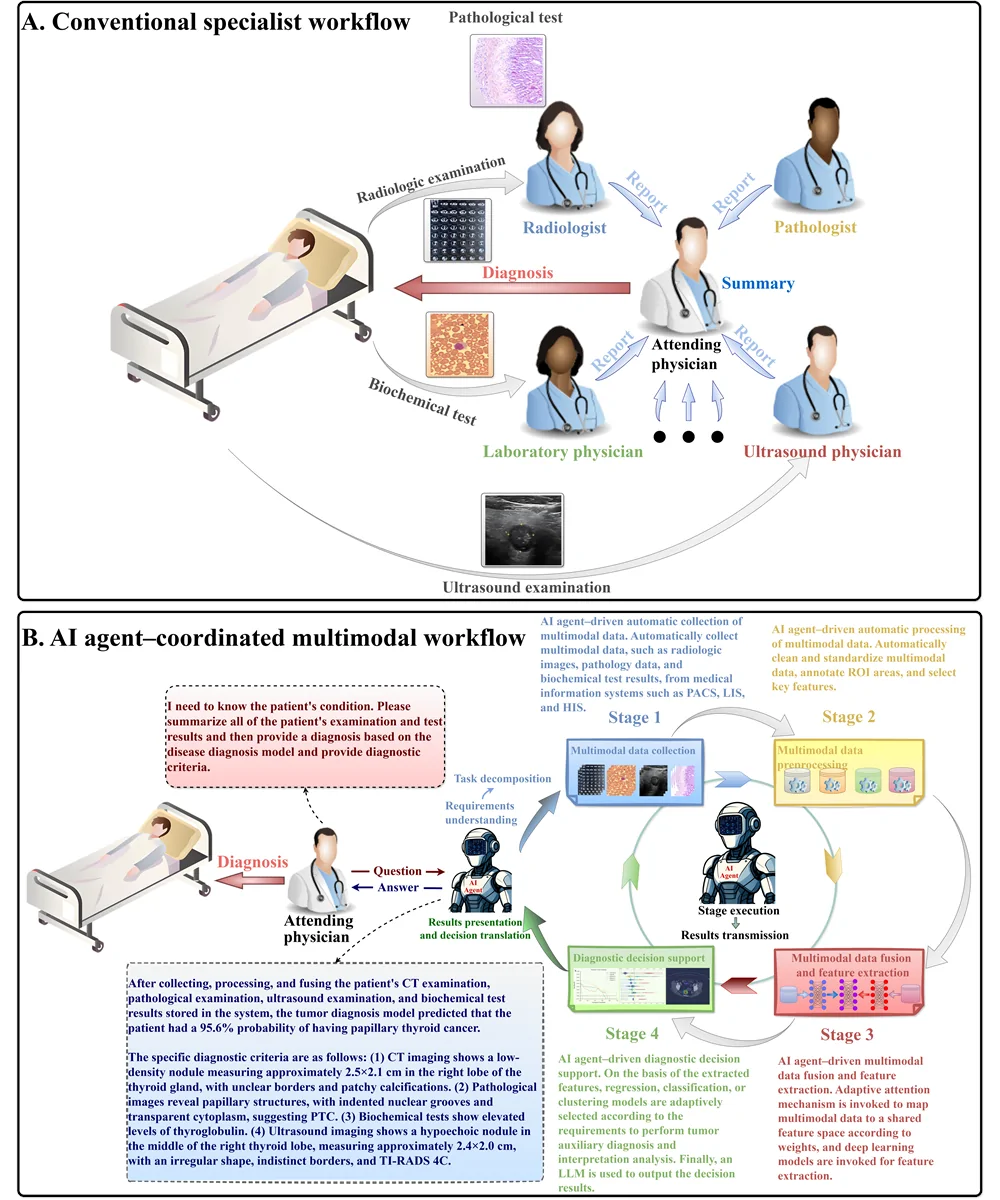
Conventional and AI agent–coordinated multimodal oncology workflows. (A) In the conventional workflow, the attending physician synthesizes modality-specific reports. (B) In the proposed agent-coordinated workflow, the agent coordinates multimodal data collection, preprocessing, fusion and feature extraction, and diagnostic decision support. CT: computed tomography; HIS: hospital information system; LIS: laboratory information system; LLM: large language model; PACS: picture archiving and communication system; PTC: papillary thyroid carcinoma; ROI: region of interest; TI-RADS 4C: Thyroid Imaging Reporting and Data System, category 4C.

**Figure 2:**
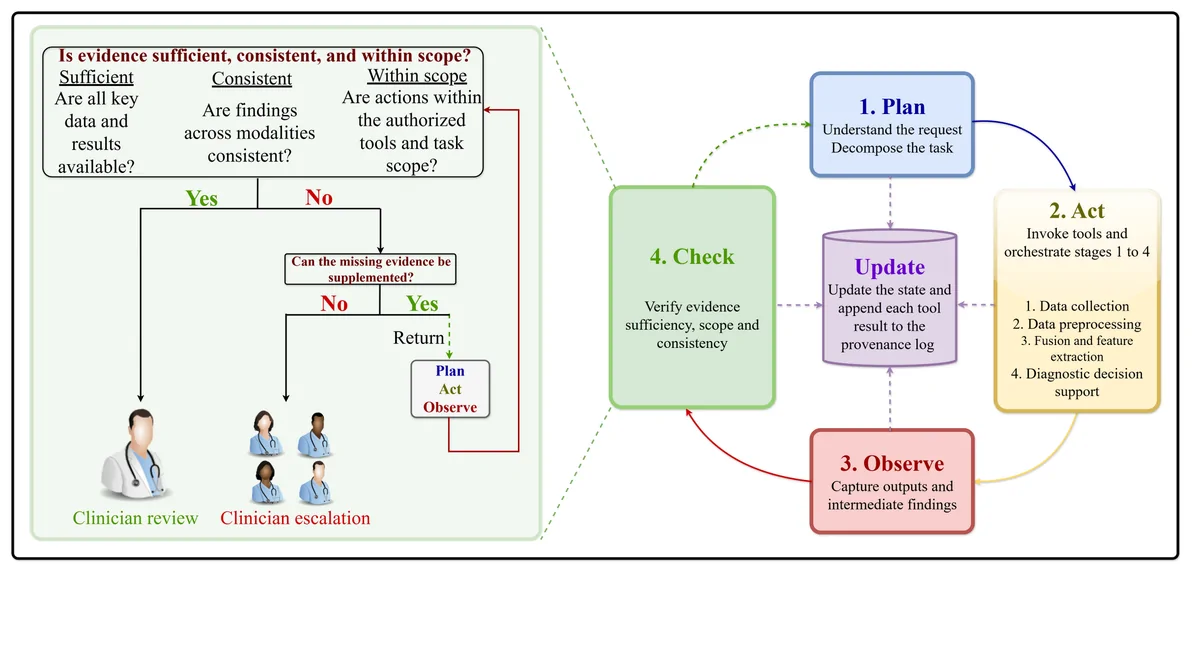
Feedback-driven orchestration and evidence-adequacy control for an AI agent in multimodal oncology diagnostics. The control architecture links plan, act, observe, update, and check. Act invokes governed tools across the 4 task stages shown in Figure 1B. Update appends each tool result to the current state and provenance log. Check evaluates whether evidence is sufficient and consistent across modalities and whether further action remains within the authorized scope. If these criteria are met, the synthesis proceeds to clinician review. Remediable gaps return the workflow to planning, whereas gaps that cannot be supplemented trigger clinician escalation.

## Multimodal Data Collection

The first stage is the governed assembly of patient-specific evidence. Relevant data may be distributed across a hospital information system (HIS), laboratory information system (LIS), picture archiving and communication system (PACS), electronic medical record (EMR), pathology archives, and genomic platforms. These systems differ in format, ontology, temporal granularity, and access control.

Component-level infrastructure studies show that integration must be engineered. A single-center oncology data supply chain linked clinical, genomic, and imaging data for more than 170,000 patients across 11 cancer types and more than 800 features per case [13]. Its extract-transform-load (ETL), natural language processing (NLP), and quality control procedures support the feasibility of a data layer but not the validation of an agent. Standards such as Health Level Seven Fast Healthcare Interoperability Resources (HL7 FHIR) and Digital Imaging and Communications in Medicine (DICOM) can reduce site-specific translation [14]. Local mapping and governance nevertheless remain necessary.

An agent could use secure interfaces to identify, retrieve, and temporally align data for a specific diagnostic question. It could connect a new scan to prior pathology, biomarker trajectories, sequencing results, and notes [9,15]. Unstructured text and patient-reported or wearable data may add context in selected settings [16]. Each input should carry its source, acquisition and update times, missingness status, and freshness threshold. Contradictory or stale records should trigger clarification rather than silent fusion. Once evidence is assembled, the next risk is whether every modality is technically fit for analysis.

## Multimodal Data Preprocessing

Multimodal preprocessing converts heterogeneous inputs into representations that downstream tools can use. Imaging varies by scanner and acquisition protocol. Digital pathology depends on tissue preparation, staining, and slide digitization [6]. Laboratory and genomic data may be incomplete or generated on different platforms, while clinical text often contains duplication and institution-specific shorthand.

A fixed workflow applies the same preprocessing sequence. An agentic workflow could select modality-specific tools according to file type, quality, and clinical purpose. It might invoke imaging quality control and segmentation or pathology stain normalization, tiling, tissue filtering, and feature extraction. Pathology foundation models provide reusable component representations [17,18], while self-supervised medical imaging models illustrate robustness and data-efficiency strategies [19]. Attribution tools such as gradient-weighted class activation mapping can support region-level inspection, although an attribution map is not itself a clinical explanation [20].

Every action should be recorded with the input source, time stamp, parser or model version, quality control result, and review status. If optical character recognition or parsing fails, the system should not fabricate a normalized record. It should retry within a bounded policy, switch to a validated alternate parser, return a partial result with an explicit missingness flag, or request human review. This provenance-aware preprocessing creates the conditions for fusion without concealing upstream uncertainty.

## Multimodal Fusion and Representation Learning

Fusion is not the mere presence of multiple data types. Imaging describes macroscopic phenotype, pathology reveals tissue architecture, molecular assays characterize biological drivers, and longitudinal records provide clinical context. Multimodal models can combine these views for characterization and risk estimation [7], but many assume that all modalities are harmonized and simultaneously available.

At the molecular level, machine learning can integrate complementary omics layers and prioritize regulatory interactions. Omidi combined RNA sequencing and microRNA sequencing profiles from 2 public cohorts [21]. The analysis included 78 adrenocortical carcinoma samples from The Cancer Genome Atlas adrenocortical carcinoma (TCGA-ACC) and 250 normal adrenal samples from the Genotype-Tissue Expression (GTEx) project, reconstructing condition-specific competing endogenous RNA networks. Random forest models performed best for predicting microRNA-messenger RNA associations, and predicted interactions were cross-referenced with TargetScan and miRTarBase. This study is an in silico systems biology module that an agent could invoke. Batch effects, cohort imbalance, cross-cohort heterogeneity, and the absence of independent or experimental validation prevent these findings from being interpreted as validation of a multimodal agent.

An agent could select a fusion strategy according to the clinical question, available modalities, and uncertainty. If pathology and imaging are available while genomic testing is pending, it could use a modality-robust model, mark molecular evidence as unavailable, and revise the synthesis later. Explainable multimodal real-world models have identified patient-level marker contributions in large cohorts and undergone independent validation [22]. These results support component-level representation and explanation but not autonomous clinical orchestration. Fusion outputs must therefore pass an evidence-adequacy check before decision support. The evidence-adequacy gate and its return-or-escalate logic are shown in Figure 2.

## Diagnostic Decision Support

The final stage translates analyzed evidence into a structured, reviewable diagnostic summary. The purpose is not autonomous diagnosis or treatment selection. It is to state the supported findings, unresolved conflicts, missing evidence, uncertainty, and provenance in a form that clinicians can audit.

A recent feasibility study evaluated Gemini 2.5 Pro (Alphabet Inc) and ChatGPT 4o (OpenAI) on 80 Turkish-language prostate-specific membrane antigen positron emission tomography–computed tomography (PSMA PET-CT) reports [23]. The prompts incorporated the American Joint Committee on Cancer (AJCC) staging, the Chemohormonal Therapy Versus Androgen Ablation Randomized Trial for Extensive Disease (CHAARTED) criteria, and few-shot examples. The models assigned T, N, and M categories and disease volume classes, with overall task accuracies of 93.8% and 91.3%, respectively. Errors clustered around equivocal findings and different interpretive thresholds, including overstaging of ambiguous lesions. The single-center design, 1 expert reference standard, and exploratory sample of 80 reports limit generalizability. The study supports an LLM-based report-to-staging component that requires expert oversight; it does not validate a complete AI agent.

Other component studies illustrate evaluable end points. Multimodal oncology chatbots have been tested on image-containing clinical cases [24]. Explanation types can alter physician trust and diagnostic performance [25]. On the contrary, fabricated details can induce hallucinated clinical elaboration despite simple mitigation attempts [26]. Agent evaluation should therefore include tool selection, evidence citation, contradiction handling, safe refusal, calibration, workflow burden, and final conclusion quality rather than fluency alone.

In practice, an agent might prepare a multidisciplinary team (MDT) case by linking a suspicious lung lesion to pathology, molecular markers, smoking history, and prior imaging. It could rank competing diagnoses, identify a missing confirmatory test, and provide an evidence-linked summary. Proposed MDT assistance and randomized source-attribution studies support case preparation and verifiable retrieval as useful components [27,28]. Clinicians must retain authority over the final judgment, with explicit responsibility assigned across users, institutions, and manufacturers [29].

## Challenges and Future Directions

### Limits of the Current Evidence

The evidence base remains uneven. Most cited studies validate models, infrastructure, or simulated workflows rather than prospectively deployed agentic systems. The following implementation requirements should therefore be read as a translational agenda and not as established clinical effectiveness.

### Data Quality and Interoperability

Interoperability does not guarantee semantic correctness. A deployable system must map local fields to versioned ontologies, record lineage and freshness, and quantify missingness. It must also test performance across scanners, laboratories, institutions, demographic groups, and unavailable-modality patterns [13,14,19]. External validation and subgroup analysis remain necessary because an agent can amplify errors introduced by any upstream component.

### Operational Resilience and Graceful Degradation

Real-time MDT use requires an explicit latency budget for retrieval, preprocessing, inference, and rendering. Institutions may precompute stable features, use asynchronous tasks, or choose smaller local models when the expected benefit does not justify the delay. Time-outs should activate circuit breakers rather than indefinite retries. A parser or API failure should trigger a typed error and one of the following governed responses: a bounded retry, use of a validated alternate tool, a partial-result flag, or abstention with human escalation. The audit record should preserve the failed attempt and the fallback path. This fallback logic corresponds to the supplementation and clinician-escalation branches in Figure 2.

### Knowledge Life Cycle and Guideline Synchronization

Oncology guidelines and drug knowledge change frequently. Retrieval stores should preserve source dates, jurisdiction, guideline version, and effective period. Updates require staged ingestion, regression tests against reference cases, comparison with the previous version, canary deployment, and rollback criteria. A response should identify the version used and warn when local policy conflicts with an external guideline. This reduces, but cannot eliminate, legacy hallucinations.

### Evaluation and Clinical Integration

Evaluation should follow the clinical workflow and the evidence label. Component metrics include extraction accuracy, calibration, source verifiability, latency, throughput, and failure rate. Agent-level evaluation additionally requires plan appropriateness, tool-use accuracy, recovery from failed steps, contradiction resolution, abstention, clinician workload, and patient safety end points [11,24-28]. Prospective studies should compare the full human-agent workflow with current practice and not only the agent with a static model.

### Governance, Cost, and Institutional Adoption

Deployment also depends on computing cost, local infrastructure, cybersecurity, procurement, workflow fit, regulatory status, reimbursement, and institutional adoption. Trustworthy AI principles require fairness, traceability, usability, robustness, and explainability [12]. Precision oncology governance should add access control, deidentification, audit logging, incident response, continuous monitoring, model and knowledge base change control, and explicit accountability [29,30]. These controls are prerequisites for a clinical service and not evidence that the service improves outcomes.

Table 1 summarizes the current evidence supporting AI agents in oncology diagnostics and the limitations that constrain their interpretation and clinical use.

**Table 1:** Evidence supporting and delimiting AI agents in oncology diagnostics.

Evidence domains	Study design or setting	Main empirical finding	Agentic relevance and boundary
Agent-level oncology decision support	External evaluation on 20 realistic multimodal oncology cases [11]	Reported 87.5% correct tool use, 91% correct clinical conclusions, and 75.5% accurate guideline citation	Agent-level evidence from simulated cases; a useful benchmark but not prospective clinical validation
Real-world multimodal infrastructure	Single-center data supply chain covering >170,000 patients, 11 cancer types, and >800 features per case [13]	Integrated clinical, genomic, and imaging data with extract-transform-load, natural language processing, quality control, and continuously updated records	Infrastructure evidence; supports a callable data layer but does not evaluate agent planning or clinical benefit
Multimodal oncology chatbot benchmarking	Case-based evaluation of multimodal chatbots on 79 image-containing oncology cases [24]	Demonstrated that image-text oncology reasoning can be measured with case-level accuracy	Component evidence; a chatbot benchmark does not establish workflow orchestration or safety
Large language model–assisted prostate cancer staging from unstructured imaging reports	Single-center feasibility study of 80 Turkish-language prostate-specific membrane antigen positron emission tomography–computed tomography reports with embedded American Joint Committee on Cancer and Chemohormonal Therapy Versus Androgen Ablation Randomized Trial for Extensive Disease criteria and few-shot prompting [23]	Overall task accuracy was 93.8% for Gemini 2.5 Pro and 91.3% for ChatGPT 4o; errors clustered around equivocal findings	Component evidence; supports a report-to-staging module requiring expert oversight and not a complete agent
Machine learning integration of molecular regulatory networks	In silico integration of 78 The Cancer Genome Atlas adrenocortical carcinoma tumors and 250 normal adrenal samples from the Genotype-Tissue Expression project using RNA and microRNA sequencing [21]	Random forest best predicted microRNA-messenger RNA associations; predictions were cross-referenced with TargetScan and miRTarBase.	Specialized analytic module; cohort imbalance, batch effects, and the absence of independent or experimental validation limit inference
Tumor MDT^a^ workflow	Workflow-focused proposal for AI assistance in tumor MDTs [27]	Identified opportunities for case preparation, synthesis of unstructured data, and rationale capture	Prospective workflow proposition; final decisions remain clinician led and require comparative evaluation
Source-attributed uro-oncology decision support	Randomized reader evaluation of a retrieval-augmented uro-oncology chatbot [28]	Improved recommendation correctness, source attribution, and source verifiability compared with a general chatbot	Component evidence; supports governed retrieval and verifiable sources and not full agent autonomy
Explainable multimodal real-world oncology AI	Multimodal model in 15,726 patients across 38 solid cancer types with external validation in 3288 patients with lung cancer [22]	Estimated patient-level marker contributions and prognostic interactions	Model-level evidence; supports reusable prediction and explanation tools with external validity boundaries
Hallucination and safety	Multimodel simulation of adversarial hallucination during clinical decision support [26]	Fabricated details induced false elaboration despite simple mitigation prompts	Safety evidence supporting contradiction checks, retrieval constraints, abstention, and human escalation
Interoperability	Clinical interoperability analysis across the AI data life cycle [14]	Emphasized standardized information exchange for data entry, processing, sharing, replication, and analytics	Infrastructure evidence; local semantic mapping and provenance remain necessary
Governance, liability, and implementation	Consensus trustworthy AI guidance, liability analysis, and precision oncology implementation review [12,29,30]	Identified requirements for fairness, traceability, usability, robustness, explainability, responsibility, safety, governance, cost, and harmonization	Cross-cutting requirements; governance controls enable evaluation but are not evidence of clinical effectiveness

^a^MDT: multidisciplinary team.

### Conclusions

AI agents should be understood as auditable, feedback-driven orchestration systems rather than as another name for multimodal models or RAG. Oncology-specific studies now support selected agent-level, component-level, and infrastructure capabilities, including tool use, report-to-staging, multimodal modeling, and source attribution. However, these findings do not establish the safety or benefit of autonomous diagnosis. The defensible near-term goal is a resilient human-agent workflow that exposes provenance, uncertainty, failures, guideline versions, and escalation decisions. Prospective comparison with current clinical practice is required before broader implementation claims are justified.

## Abbreviations

AJCC: American Joint Committee on Cancer
CHAARTED: Chemohormonal Therapy Versus Androgen Ablation Randomized Trial for Extensive Disease
DICOM: Digital Imaging and Communications in Medicine
EMR: electronic medical record
ETL: extract-transform-load
GLOBOCAN: Global Cancer Observatory
GTEx: Genotype-Tissue Expression
HIS: hospital information system
HL7 FHIR: Health Level Seven Fast Healthcare Interoperability Resources
LIS: laboratory information system
LLM: large language model
MDT: multidisciplinary team
NLP: natural language processing
PACS: picture archiving and communication system
PSMA PET-CT: prostate-specific membrane antigen positron emission tomography–computed tomography
RAG: retrieval-augmented generation
TCGA-ACC: The Cancer Genome Atlas adrenocortical carcinoma

## Data Availability

Data sharing is not applicable to this article as no datasets were generated or analyzed during this study.
